# Recombination Analysis of Non-Poliovirus Members of the Enterovirus C Species: Restriction of Recombination Events to Members of the Same 3DPol Cluster

**DOI:** 10.3390/v12070706

**Published:** 2020-06-30

**Authors:** Lieke Brouwer, Kimberley S.M. Benschop, Dung Nguyen, Everlyn Kamau, Dasja Pajkrt, Peter Simmonds, Katja C. Wolthers

**Affiliations:** 1 Department of Medical Microbiology, Academic Medical Center (AMC), Amsterdam University Medical Centers (Amsterdam UMC), 1105AZ Amsterdam, The Netherlands; k.c.wolthers@amsterdamumc.nl; 2 Centre for Infectious Disease Control, National Institute for Public Health and the Environment (RIVM), 3721MA Bilthoven, The Netherlands; kim.benschop@rivm.nl; 3 Nuffield Department of Medicine, University of Oxford, Oxford OX1 3SY, UK; dung.nguyen@ndm.ox.ac.uk (D.N.); everlyn.kamau@lmh.ox.ac.uk (E.K.); peter.simmonds@ndm.ox.ac.uk (P.S.); 4 Department of Pediatric Infectious Diseases, Academic Medical Center (AMC), Amsterdam University Medical Centers (Amsterdam UMC), 1105AZ Amsterdam, The Netherlands; d.pajkrt@amsterdamumc.nl

**Keywords:** Enterovirus, recombination

## Abstract

Enteroviruses (EVs) are highly prevalent viruses worldwide. Recombination is known to occur frequently in EVs belonging to species *Enterovirus A*, *Enterovirus B*, and *Enterovirus C*. Although many recombinant vaccine-derived poliovirus (VDPV) strains have been reported, our knowledge on recombination in non-polio EVs in the species *Enterovirus C* is limited. Here, we combined a dataset consisting of 11 newly generated full-length *Enterovirus C* sequences and 180 publicly available sequences to study recombination dynamics in non-polio EVs. To identify recombination patterns, maximum likelihood phylogenetic trees of different genomic regions were constructed, and segregation analyses were performed. Recombination was observed between members of the same 3DPol cluster, but was rarely observed between members of different clusters. We hypothesize that this restriction may have arisen through their different compartmentalization in respiratory and enteric tracts related to differences in cellular tropisms so that the opportunity to recombine may not be available.

## 1. Introduction

Enteroviruses (EVs) are single stranded positive-sense RNA viruses in the family of *Picornaviridae*. Upon infection, EVs can cause a wide variety of symptoms and disease outcomes, ranging from mild respiratory or gastro-intestinal symptoms to meningitis, encephalitis, and acute flaccid paralysis [1]. All currently known EVs belong to one of 15 species (*Enterovirus A*-*Enterovirus L* and *Rhinovirus A*-*Rhinovirus C*), with *Enterovirus A*–*Enterovirus D* and all three rhinovirus species known to infect humans [2]. The EV genome is approximately 7500 nucleotides (nts) in length. It encodes a polyprotein consisting of a P1, P2, and P3 region, flanked by untranslated regions (UTRs) at both the 5′ and 3′ end. The P1 region is further cleaved into 4 structural proteins that make up the viral capsid (VP1-VP4), while the P2 and P3 region are further cleaved into non-structural proteins (2A - 2C and 3A - 3D, respectively) required for virus replication.

Recombination is often observed within members of species *Enterovirus A*, *Enterovirus B*, and *Enterovirus C*, but recombination events are rare in *Enterovirus D* [3]. Recombination events in EVs are almost exclusively detected at the edges of the structural P1 region, or within the non-structural 5′UTR, P2 or P3 region [4,5]. Though recombination has been shown to occur across the whole genome, not all recombinants may be viable, and there would thus be selection of recombinants with breakpoints in these specific locations [6]. 

Recombination is usually demonstrated by showing phylogeny violations; dissimilar clustering of viral strains between evolutionary trees constructed from nt sequences of different regions of the genome [7,8,9,10,11,12,13,14]. Recombinant forms (RFs) can then be assigned based on clustering of the strains in the nonstructural region [9,10,12,13,14], and breakpoints can be detected by performing bootscanning [7,8,11]. Among members of *Enterovirus A*, recombination is common, although EV-A71 shows lower recombination rates compared to other types in this species [13,15,16]. Recombination rates in *Enterovirus B* are high [4] and a vast number of recombinant forms have been identified in this species [9,10,12]. Among members of *Enterovirus C*, three genetic subgroups have been described (Ca, Cb, and Cc) between which recombination appears to be restricted, although there is frequent recombination within these subgroups [7]. In the current study, for clarity, we will refer to these subgroups as subspecies C1, C2, and C3, respectively. Recombinant forms of the three serotypes of poliovirus in subspecies C3—and specifically their live attenuated vaccine strains—are frequently reported, and can contain fragments of coxsackievirus A11 (CVA11), CVA13, CVA17, and CVA20 strains, all members of subspecies C3 [17,18,19]. Outbreaks of poliomyelitis caused by these vaccine derived polioviruses (VDPVs) have been reported in several countries where the live attenuated vaccine is used [20,21,22]. Furthermore, it has been shown that in vitro constructed recombinants of PV and other subspecies C3 strains are viable in cell culture [23]. Though PVs were highly endemic worldwide, the other *Enterovirus C* types are mainly endemic in African countries whereas they are rarely detected in Europe, Asia, and North America [24,25,26,27,28,29,30,31,32]. As a result, only a small number of sequences are available for members of *Enterovirus C*, compared to *Enterovirus A* or *Enterovirus B*, and few recombination analyses have been conducted on this species. 

In the current study, we used full length sequences obtained from *Enterovirus C* positive samples from a previous study [24] and publicly available *Enterovirus C* sequences to study recombination dynamics within this species.

## 2. Materials and Methods 

### 2.1. Sample Processing and Full Length Sequencing

Stool samples positive for *Enterovirus C* (*n* = 41) by VP1 sequencing from a previous study [24] were selected for full length sequencing. For viral RNA extraction, the QIAamp Viral RNA Mini Kit (Qiagen, Hilden, Germany) was used according to manufacturer’s instructions. For all samples, 4 µL of extracted RNA was added to 1 µL of random primer (N9). The mixture was incubated at 65 °C for 5 min. 5 µL of Superscript First Strand mastermix (2 µL 5X first strand buffer, 0.75 µL water, 1.25 µL 0.1 M dNTP, 0.5 µL 0.1 M DTT, 0.5 µL Superscript III RT (Thermo Fischer, Waltham, USA)) was added and the samples were incubated at 42 °C for 60 min. 5 µL of Sequenase Mix 1 (1 µL 5x Sequanase buffer (Thermo Fischer), 3.85 µL water, 0.15 µL Sequenase Enzyme (Thermo Fischer)) was added and the samples were incubated at 37 °C for 8 min. 0.6 µL of Sequenase Mix 2 (0.45 µL Sequenase dilution buffer, 0.15 µL Sequenase enzyme) was added and the samples were incubated at 37 °C for 8 min. The obtained double stranded cDNA was purified using AMPure XP beads (Beckman Coulter, Brea, USA) according to manufacturers’ instructions. The DNA concentration was measured by Qubit (Thermo Fischer) (according to manufacturer’s instructions), and all samples were normalized to 0.2 ng/µL. For tagmentation and library preparation, the Nextera XT DNA Library Preparation Kit (Illumina, San Diego, USA) was used according to manufacturers’ instructions, and samples were purified using AMPure XP beads. The Qubit cDNA kit was used to quantify the libraries, and the size distribution was determined using the Agilent 2200 TapeStation Bioanalyzer. Libraries were normalized to 1.6 ng/µL (4 nM) with Tris-Cl 10mM pH 8.5 with 0.1% Tween. The normalized libraries were incubated at 95 °C for 5 min while shaking at 1800 rpm. 5 µL of each library was transferred to a single low bind tube. 15 µL of the DNA pool was added to 15 µL of 0.2M NaOH to denature the DNA into single strands. The denatured DNA pool was added to pre-chilled Hybridization Buffer (HT1) to obtain 600 µL of 16pM DNA. 6 µL of 12.5 pM Phix was added to 594 µL of the denatured pool for a 1% PhiX concentration. Sequencing was performed using the MiSeq Illumina cartridge and sequencer. The raw data were processed using GenomeDetective version 1.111 [33]. The obtained sequences were deposited in the DDBJ/GenBank/EMBL database under accession numbers MN914196-MN914206.

### 2.2. Database Construction

A database was constructed comprising all our study sequences that included at least the entire VP1 through 3DPol genomic regions (nt positions 2457–7334 based on reference strain CVA21 Kuykendall, accession number AF546702), and all unique non-polio *Enterovirus C* sequences of at least 7000 nts in length available in the DDBJ/GenBank/EMBL database (April 2019). Strains that were reported as clones and one EV-C99 strain (MH144606) that was found to contain sequencing artifacts were excluded. An Echovirus 1 strain (AF029859) was added as an outgroup for the construction of phylogenetic trees. A multiple sequence alignment was made using Mafft version 7.429 [34]. Information on year of detection, country of detection, and sample type (e.g., stool or respiratory sample) was extracted from GenBank for all sequences. If information on a sequence was not available in GenBank, it was retrieved from the original article.

### 2.3. Detecting Phylogenetic Violation

Phylogenetic trees of the VP1 (nt positions 2457–3350, reference genome AF546702), 2C (nt positions 4089–5072), and 3DPol (nt positions 5952–7334) genomic region were constructed using the maximum likelihood (ML) method with the general time reversible (GTR) + Gamma substitution model in RAxML version 8.2.10 [35]. The trees were visualized using the ggtree package version 1.16.4 [36] in R version 3.6 [37]. Log-likelihood values of the three topologies were evaluated for each of the three alignments (VP1, 2C and 3DPol) using the Shimodaira-Hasegawa method [38] implemented in RaxML version 8.2.10 [35]. 

Segregation analyses were performed using the TreeOrder scan function in SSE version 1.3 [35] using a window of 300 nts and steps of 24 nts. Segregation analyses were performed on the complete dataset for segregation into subspecies, and on each of the subspecies C1, C2, and C3 for segregation into types.

### 2.4. Plotting p-Distance and Determining Recombinant Forms

Pairwise p-distances between the VP1 and 3DPol regions of all sequences were extracted using SSE version 1.3. Histograms of the 3DPol pairwise p-distances were constructed in R version 3.6. Plots mapping VP1 and 3DPol p-distance for each sequence pair were constructed for each subspecies (C1, C2 and C3). 

Clusters in the 3DPol genomic region were defined based on the pairwise p-distances in this region. Recombinant forms were assigned using a sequence p-distance threshold of 0.06–0.12 in the 3DPol region, determined through analysis of the distance distribution; these thresholds are comparable to those estimated in previous studies of EV-A71 and E30 [12,13].

## 3. Results

**Figure 1:**
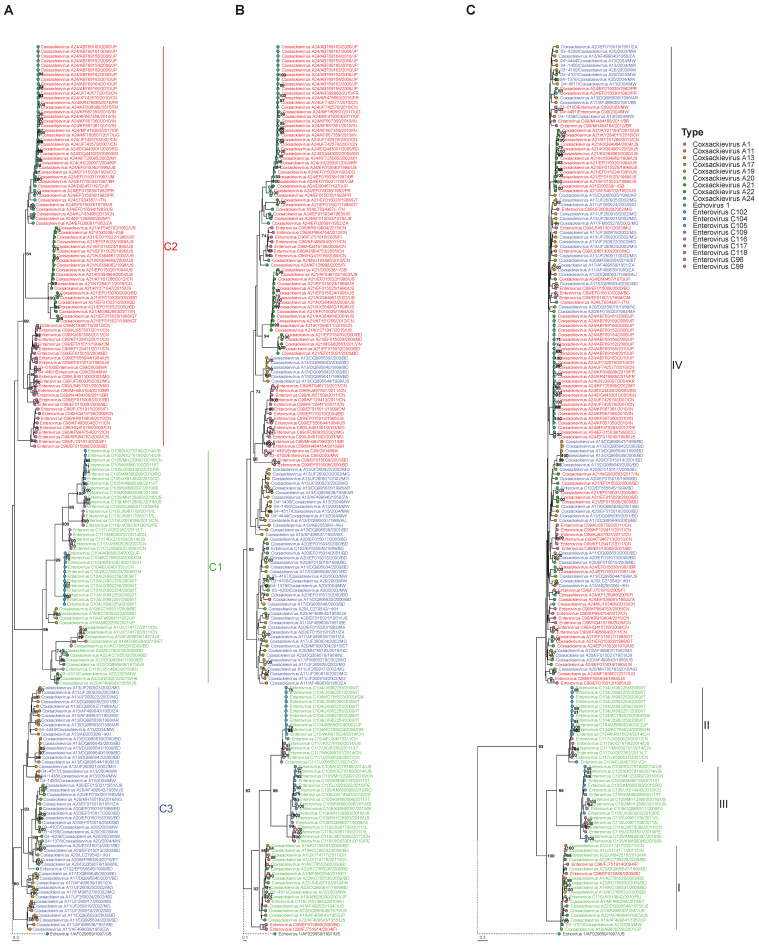
Phylogenetic trees (Maximum Likelihood method with GTR-Gamma nt substitution model) of the VP1 (**A**), 2C (**B**) and 3DPol (**C**) genomic regions containing all study strains and *Enterovirus C* strains retrieved from GenBank. GenBank extracted strains are named as type/accession number/two-letter country code of country of detection/ year of detection. Study strains are named with their studynumber including the year of isolation (P02-XXXX, 2002; P03-XXXX, 2003; P04-XXXX, 2004). The subspecies and subgroups (C1, C2, C3) are marked in the VP1 tree and strain names are colored accordingly. Tips are colored according to type. The identified clusters (I, II, III, IV) are marked in the 3DPol tree. Bootstrap values >70% are shown. The length of the branch of the outgroup (Echovirus 1/AF029859/1997/US, branch shown with dotted line) was adjusted to fit the figure margins.

Sequences including the full VP1 through 3DPol sequence were obtained from 11 of 41 study strains and were included for further analyses. Based on their clustering in the phylogenetic tree of the VP1 region (Figure 1A), the study strains were typed as EV-C99 (03-1000, 04-4491), CVA13 (04-4444, 04-4517, 04-1438, 04-1450), CVA20 (03-4166, 03-4107, 04-1378, 03-4209), and CVA22 (03-4101). A total of 180 non-polio *Enterovirus C* sequences of more than 7000 base pairs were extracted from GenBank (April 2019). The complete database contained 191 sequences. Phylogenetic trees were constructed from the VP1, 2C, and 3DPol region. The Shimodaira-Hasegawa tests showed that VP1, 2C, and 3DPol alignments were significantly better explained by their respective topologies than by those of other genomic regions (*p* < 0.01), consistent with the occurrence of recombination events.

### 3.1. Segregation of Sequences in the VP1 Genomic Region

Based on their clustering in the VP1 coding region, *Enterovirus C* strains can be divided into three subspecies, which we named here C1 (CVA-1, -19,-22 and EV-C104, -C105, -C109, -C116, -C117 and -C118), C2 (CVA-21, -24 and EV-C96 and –C99) and C3 (CVA11, -13, -17, -20 and EV-C102) for clarity (previously named Ca, Cb, and Cc, respectively [7]). In addition to the clustering in these subspecies, all strains segregated into type-specific clusters in the VP1 coding region, except for the CVA22 strains and one EV-C116 strain, which clustered together (Figure 1A). 

### 3.2. Segregation of Sequences in the 2C Genomic Region

In the phylogenetic tree of the 2C coding region (Figure 1B), six CVA13 strains clustered with subspecies C2, and two EV-C96 strains clustered within subspecies C1 (all reported before [7]), while all other strains remained in their subspecies-specific clusters. Segregation into type-specific clusters was violated for all types, except for EV-C105, EV-C109, EV-C118, EV-C104, and EV-C117 (in subgroup C1). 

### 3.3. Segregation of Sequences in the 3DPol Genomic Region

**Figure 2:**
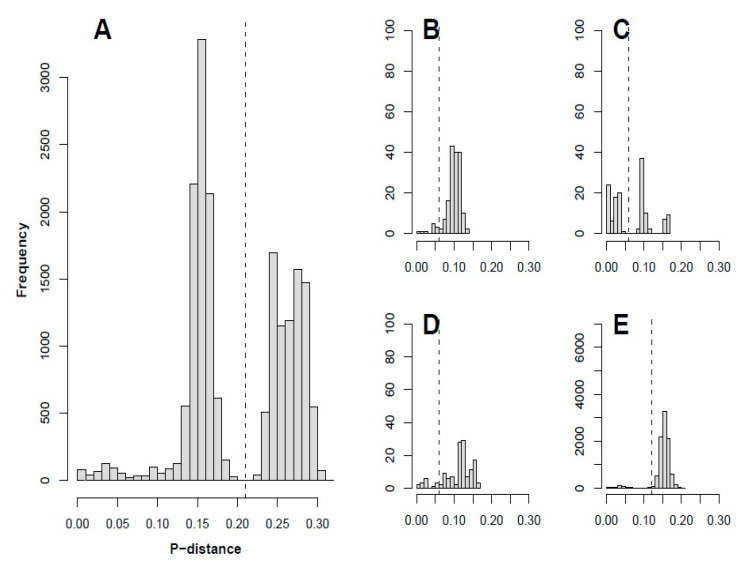
Histograms of pairwise p-distances in the 3DPol region of (**A**) All sequences and (**B**,**C**,**D**,**E**) Sequences within cluster I, II, III, and IV, respectively.

Clusters were defined based on the pairwise p-distances in the 3DPol coding region in the complete dataset, which showed a bimodal distribution (Figure 2A). The two peaks represented intra- and inter cluster distances. A cut-off p-distance of 0.21 was set in between the two local maxima, separating the sequences into four distinct clusters, as shown in the phylogenetic tree (Figure 1C). This approach has been applied previously in defining different VP1-based clades in parechoviruses [39,40]. Strains belonging to subspecies C2 and C3 grouped together in cluster IV, while strains belonging to types CVA1, CVA19, CVA22, and EV-C116 in subspecies C1 and two EV-C96 strains formed cluster I. Strains belonging to types EV-C104 and EV-C117 formed cluster II while strains in EV-C105, EV-C109, and EV-C118 formed cluster III. The definition of these clusters was further supported by mapping the pairwise VP1 and 3DPol p-distances (Figure S1). Violation of type-specific clustering occurred across the phylogeny, although there were two clusters of only CVA21 and only CVA24 sequences, respectively.

### 3.4. Segregation of Sequences Across the Genome

**Figure 3:**
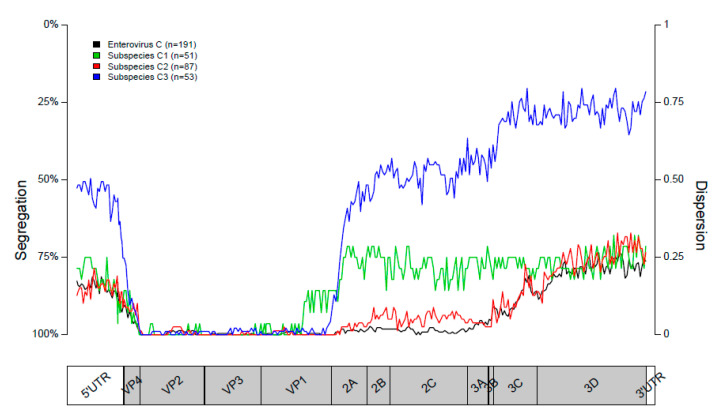
Segregation plots of all sequences, showing the segregation into subspecies (in black) and of sequences belonging to subspecies C1, C2, and C3 (green, red, and blue, respectively) showing the segregation into types. The position in the genome is shown on the x-axis. The dispersion value is shown on the right x-axis. The percentage of segregation (inverse of the dispersion) is shown on the right y-axis.

Segregation of the sequences into subspecies was close to perfect in the structural P1 region of the genome. Segregation scores remained high throughout the P2 region but were lower in the 5′UTR and P3 region (Figure 3). Segregation into type-specific clusters in subspecies C1 decreased instantly at the start of the P2 region. In subspecies C2, segregation remained high throughout the P2 region but decreased in the P3 region. In subspecies C3, segregation vastly decreased at the start of the P2 region and dropped even further in the P3 region (Figure 3). These results are in line with the clustering of sequences in the phylogenetic trees constructed for the VP1, 2C, and 3DPol coding regions in the P1, P2, and P3 regions, respectively.

### 3.5. Recombinant Forms

P-distance cut-offs for defining recombinant forms (RFs) were based on the p-distance distribution in the 3DPol region (Figure 2B–E). The cut-off for cluster I, II, and III was set at a p-distance of 0.06. For cluster IV the cut-off was set at 0.12. A total of 89 RFs were identified in the current dataset. Most of the recombinant forms were represented by single strains, while 26 RFs each comprised multiple strains. The three RFs that occurred most frequently were in EV-C104/EV-C117, CVA21, and CVA24 strains (12, 14, and 25 strains with the same RF, respectively). One RF was shared by three CVA20 study strains (04-1378, 03-4166, 03-4107), and another was shared by two EV-C99 study strains (03-1000, 04-4491). 

### 3.6. Clustering Associated with Site of Origin

The viral strains were categorized as derived from gastro-intestinal, respiratory, or ocular samples, based on information on sample type (e.g., stool sample or throat swab) available in GenBank. All of the strains with known sample type information in 3DPol clusters I and IV were derived from gastro-intestinal samples, except for a cluster of CVA21 strains, which were mainly of respiratory origin, and a cluster of CVA24 strains, which were mainly of ocular origin. These CVA21 and CVA24 clusters stayed monophyletic throughout the genome, except for one CVA-24 strain from Uganda, which was closest to a Nigerian CVA20 strain in its 3DPol region. All of the strains in 3DPol clusters II and III with sample type information were of respiratory origin, except for an EV-C105 strain, which was isolated from a stool sample [41] (Figure S2).

## 4. Discussion

In the current study, we used a set of full-length sequences extracted from the DDBJ/GenBank/EMBL database and newly generated sequences from study samples to investigate recombination dynamics within species *Enterovirus C*.

In line with other studies [8,17], we see high levels of phylogenetic violation in trees based on different regions of the genome, indicating frequent recombination within *Enterovirus C*. This is further supported by the low extent of segregation in the non-coding and non-structural regions of the genome. The sharp decreases in segregation at the borders of the P1 region are in line with previous reports and suggests the existence of favored recombination sites at these locations [4,7,42]. The decrease in segregation in the beginning of the P3 region implies another recombination hotspot at this location for subspecies C2 and C3. It has been shown before that recombination hotspots coincide with changes in sequence diversity; mainly the sequence diversity in the structural P1 region is higher than the diversity in the nonstructural 5′UTR, P2, P3, and 3′UTR regions [43]. 

Recombination seems to be restricted to occur within, but not between, several clusters, here named I, II, III, and IV. This is in line with reported *Enterovirus C* recombinants, which usually consist of parental genomes of types in the same cluster [5], although cluster I-II recombinants have been reported [41]. EV-C96 is the only type in our dataset that showed the ability to recombine with strains in two different clusters (cluster I and IV).

The distinct clusters within which recombination occurs may be separated due to differential tissue tropism. While viruses in subspecies C1, cluster I (C1/I) and viruses in C2/IV and C3/IV are known to infect the gastro-intestinal tract, viral strains in C1/II and C1/III are isolated almost exclusively from respiratory samples, a distinction that has been made previously for viruses in subspecies C1 [44]. This difference in tropism would imply that the groups of viruses never encounter each another, precluding recombination. The inability of the groups with a similar tissue tropism, such as C1/I and C2/IV or C3/IV, to recombine, could result from a higher sequence distance between their genomes, possibly leaving them unable to form viable viral strains upon recombination. Alternatively, these groups of viruses might infect different cell types within their target organs, thus remaining physically separated from one another.

Within cluster IV, there are two noticeable subclusters consisting of a single RF; one containing several CVA21 sequences (C2/IV), and one containing CVA24 sequences (C2/IV). The CVA24 subcluster contains almost exclusively strains isolated from conjunctival fluid in conjunctivitis patients, except for three gastro-intestinal strains from India. As has been suggested previously, these subclusters may contain a single RF due to their tissue tropism [7].

Although the genetic variety in the 3DPol region within each cluster is limited compared to the structural region of the genome, there are a vast number of RFs present, which is in line with previous reports [4,9]. The presence of different RFs in our study strains, despite some of the strains being closely related in the VP1 region, further illustrates the high rate of recombination.

The main limitation of our study is the amount of full-length sequences available, as typing is usually performed using only the (partial) VP1 sequence. As such, the available data may be biased towards specific types, geographical locations and time points originating from laboratories that conduct full length sequencing. Addition of more full-length sequences in the future that more accurately represent the diversity of circulating strains, may lead to different results and conclusions. It is therefore important that analyses on EV evolution and recombination are performed regularly and that full-length sequencing is encouraged [8]. 

In conclusion, using a dataset composed of *Enterovirus C* sequences, we have shown frequent recombination throughout the non-structural region of *Enterovirus C* strains, while the structural region seems to not be affected by recombination events, a phenomenon commonly seen for other enterovirus species. These recombination events are restricted to occur within, but not between, distinct clusters. We hypothesize that these clusters have arisen due to differences in tropism. As recombination plays an important role in EV evolution, understanding the recombination patterns of these viruses is essential in characterizing EV strains in outbreaks and to gain a deeper understanding of EV circulation. Furthermore, it provides us with valuable information for future studies on phenotypical characteristics and pathogenesis of EVs.

## Supplementary Materials

The following are available online at https://www.mdpi.com/1999-4915/12/7/706/s1, Figure S1: Pairwise VP1 and 3DPol distances, Figure S2: Clustering by site of origin.
