# Supplementary material for: Recombination Analysis of Non-Poliovirus Members of the Enterovirus C Species: Restriction of Recombination Events to Members of the Same 3DPol Cluster
Source: Viruses. 2020 Jun 30;12(7):706. doi: 10.3390/v12070706 (PMC7412211; doi:10.3390/v12070706)
Supplement: Supplementary file 1 [file viruses-12-00706-s001.zip › viruses-854216.suppl zip/viruses-854216 supplementary legend.pdf]

Figure. S1. Plots mapping the pairwise p-distances for the VP1 and 3DPol region for A All sequence pairs containing a strain in subspecies C1, B All sequence pairs containing a strain in subspecies C2, C All sequence pairs containing a strain in subspecies C3, and D, E, F All sequence pairs containing strains in C1/I, C1/II and C1/III respectively. Intra-subspecies and inter-subspecies sequence pairs are represented by different colored dots, as indicated in the legend of each plot. Dots representing non-recombined sequence pairs lie on a diagonal line from the left lower to the right upper corner. Dots that deviate substantially from this empirical line, represent sequence pairs where one or multiple recombination events have taken place. Dots representing C2-C2 and C2-C3 sequence pairs with a 3DPol-distance > 0.21 (red and blue dots in black boxes in B and C) represent sequence pairs containing an outlier EV-C96 (EF015886 or FJ751914) strain.

Figure. S2. Phylogenetic trees (Maximum Likelihood method with GTR-Gamma nt substitution model) of the VP1 (**A**), 2C (**B**) and 3DPol (**C**) regions. The strains are colored according to tropism. Bootstrap values >70% are shown. The length of the branch of the outgroup (Echovirus 1/AF029859/1997/US, branch shown with dotted line) was adjusted to fit the figure margins.
