# Supplementary figures and images for: Recombination Analysis of Non-Poliovirus Members of the Enterovirus C Species: Restriction of Recombination Events to Members of the Same 3DPol Cluster
Source: Viruses. 2020 Jun 30;12(7):706. doi: 10.3390/v12070706 (PMC7412211; doi:10.3390/v12070706)

**A**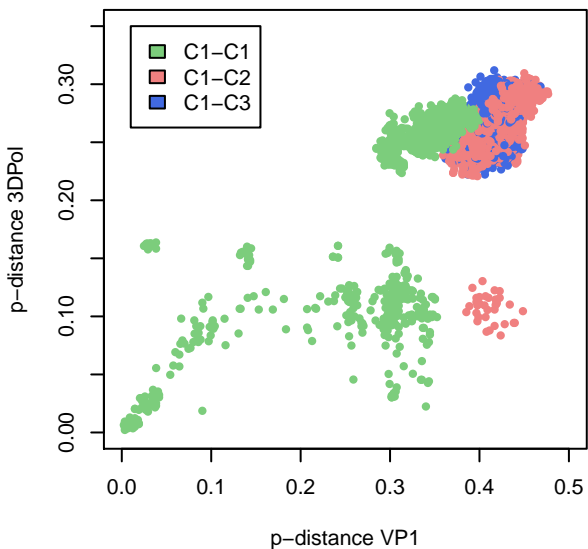**B**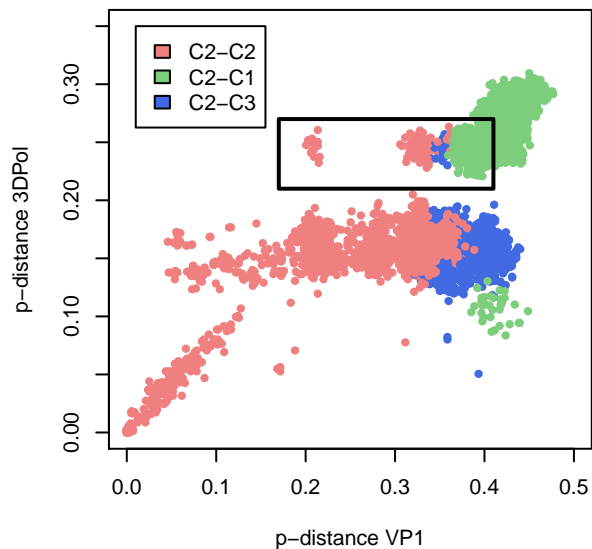**C**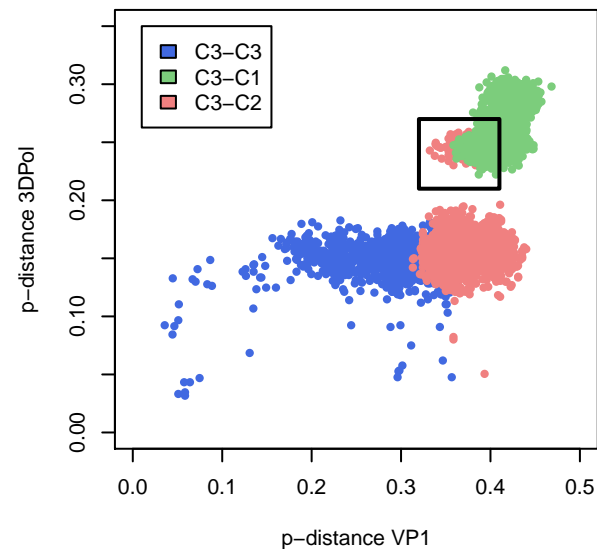**D**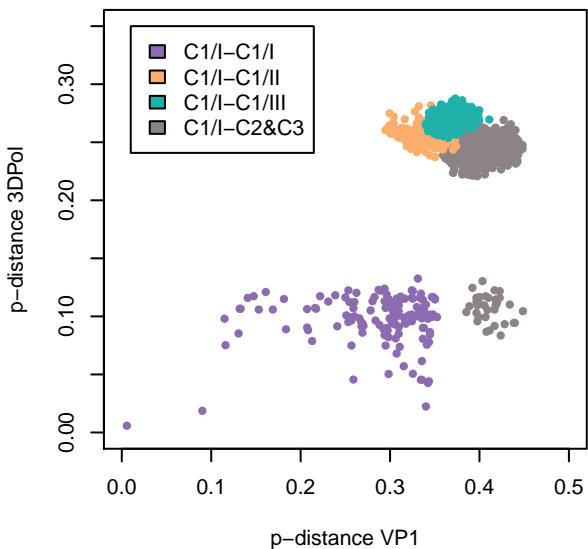**E**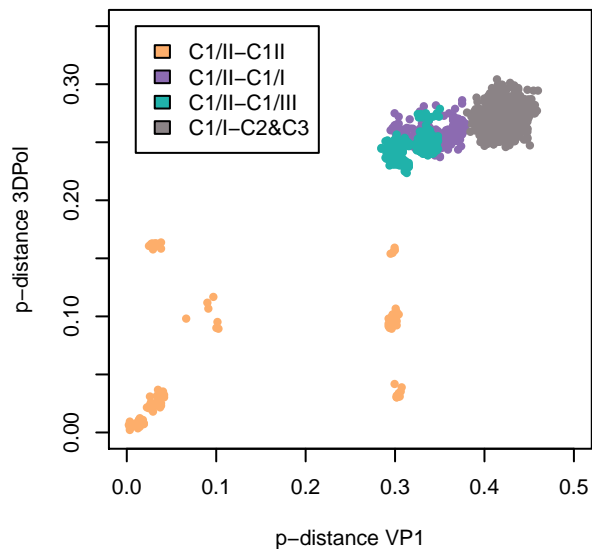**F**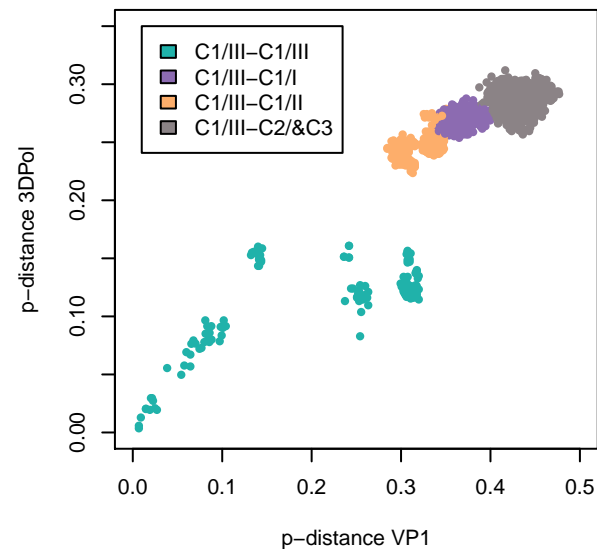

Supplement: Supplementary file 1 [file viruses-12-00706-s001.zip › viruses-854216.suppl zip/Sfig1.pdf]

# A

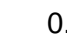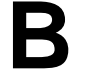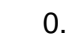

# C

# Tropism

- a Ocular
- a Gastro-intestinal
- a Respiratory
- a Unknown

Supplement: Supplementary file 1 [file viruses-12-00706-s001.zip › viruses-854216.suppl zip/Sfig2.pdf]
